# Supplementary material for: Telomerase Inhibitor TMPyP4 Alters Adhesion and Migration of Breast-Cancer Cells MCF7 and MDA-MB-231
Source: Int J Mol Sci. 2019 May 30;20(11):2670. doi: 10.3390/ijms20112670 (PMC6600420; doi:10.3390/ijms20112670)
Supplement: Supplementary file 1 [file ijms-20-02670-s001.zip › ijms-491207 sp/Supplemetary file 1, MTT - DOX conc selection and cell cycle 24 and 48h ....pptx]

## Slide 1
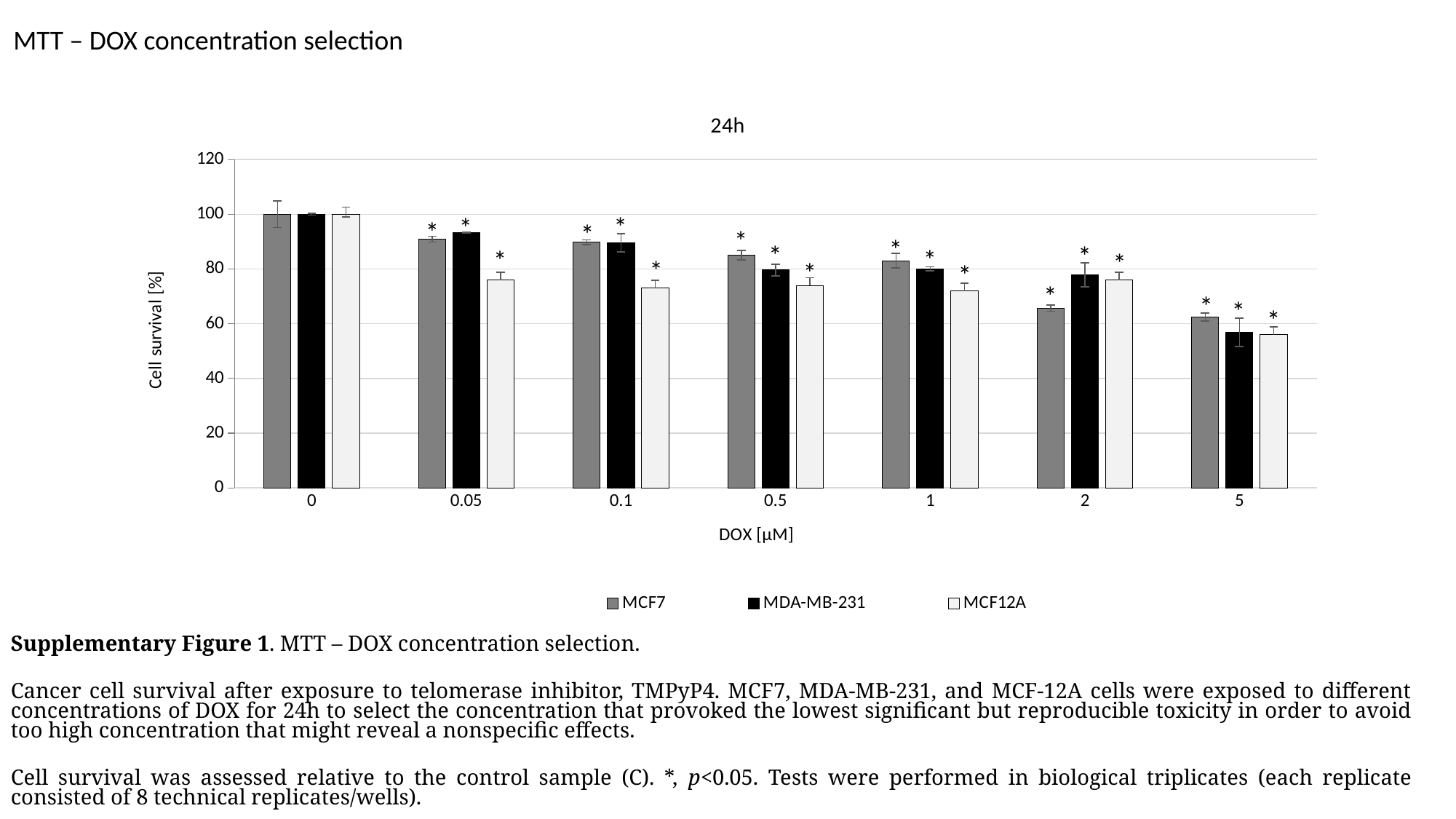

MTT – DOX concentration selection
### Chart: 24h
| Category | MCF7 | MDA-MB-231 | |
|---|---|---|---|
| 0 | 100.0 | 100.0 | 100.0 |
| 0.05 | 90.90635806010057 | 93.2739873554616 | 76.0 |
| 0.1 | 89.75510378604372 | 89.51958293937814 | 73.0 |
| 0.5 | 85.06470813234135 | 79.59573845538377 | 74.0 |
| 1 | 83.02573047621983 | 80.0532477842131 | 72.0 |
| 2 | 65.68698308593041 | 77.90817665473695 | 76.0 |
| 5 | 62.37956162687235 | 56.82863624792237 | 56.00000000000001 |*
*
*
*
*
*
*
*
*
*
*
*
*
*
*
*
*
*
Supplementary Figure 1. MTT – DOX concentration selection.
Cancer cell survival after exposure to telomerase inhibitor, TMPyP4. MCF7, MDA-MB-231, and MCF-12A cells were exposed to different concentrations of DOX for 24h to select the concentration that provoked the lowest significant but reproducible toxicity in order to avoid too high concentration that might reveal a nonspecific effects.
Cell survival was assessed relative to the control sample (C). *, p<0.05. Tests were performed in biological triplicates (each replicate consisted of 8 technical replicates/wells).

## Slide 2
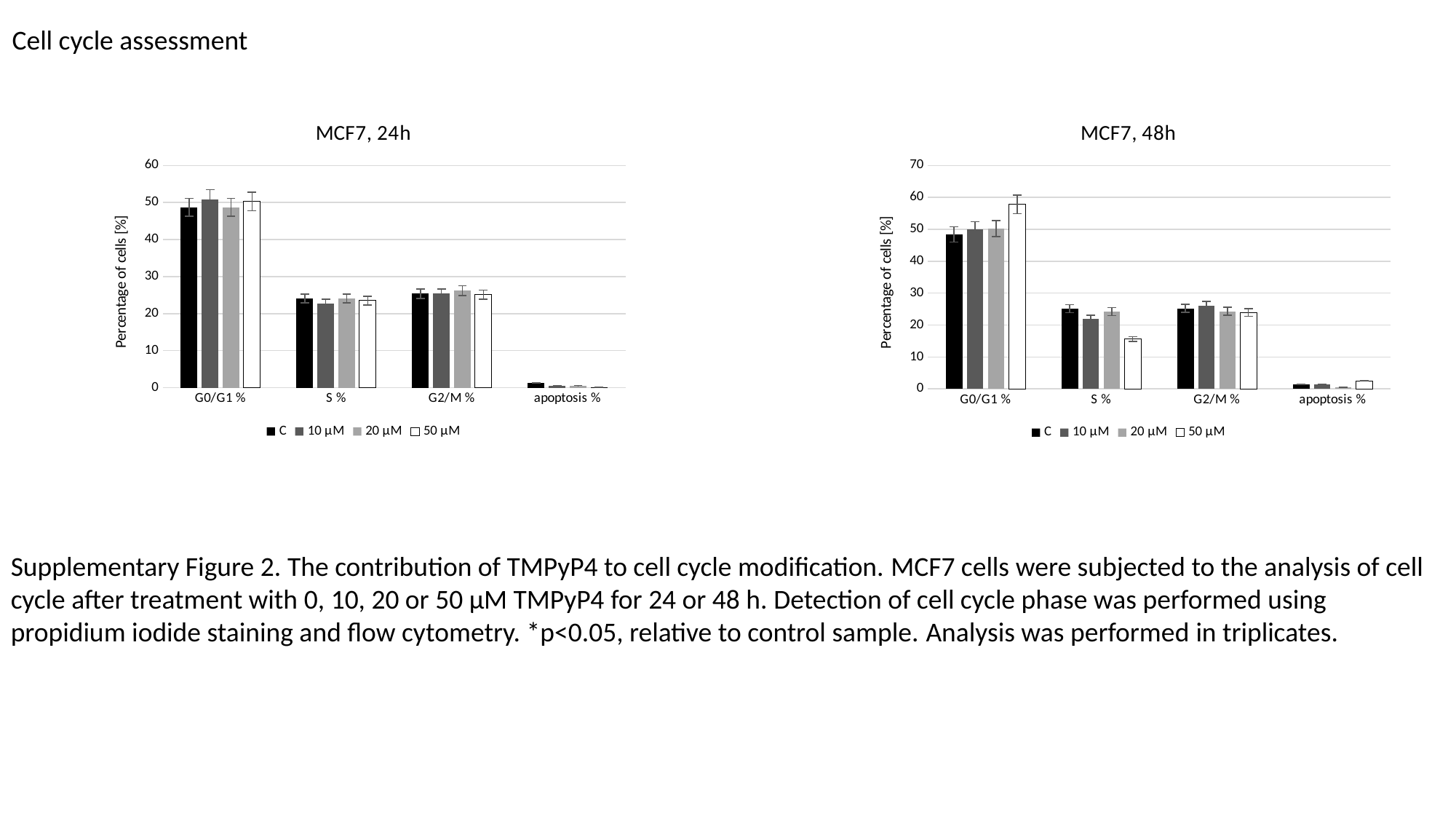

Cell cycle assessment
### Chart: MCF7, 24h
| Category | C | 10 µM | 20 µM | 50 µM |
|---|---|---|---|---|
| G0/G1 % | 48.7 | 50.9 | 48.7 | 50.3 |
| S % | 24.1 | 22.7 | 24.1 | 23.5 |
| G2/M % | 25.4 | 25.4 | 26.2 | 25.1 |
| apoptosis % | 1.3 | 0.5 | 0.6 | 0.1 |
### Chart: MCF7, 48h
| Category | C | 10 μM | 20 μM | 50 μM |
|---|---|---|---|---|
| G0/G1 % | 48.4 | 49.9 | 50.2 | 57.8 |
| S % | 25.1 | 22.0 | 24.2 | 15.6 |
| G2/M % | 25.2 | 26.1 | 24.3 | 23.9 |
| apoptosis % | 1.4 | 1.4 | 0.5 | 2.5 |Supplementary Figure 2. The contribution of TMPyP4 to cell cycle modification. MCF7 cells were subjected to the analysis of cell cycle after treatment with 0, 10, 20 or 50 µM TMPyP4 for 24 or 48 h. Detection of cell cycle phase was performed using propidium iodide staining and flow cytometry. *p<0.05, relative to control sample. Analysis was performed in triplicates.
