# Supplementary material for: Telomerase Inhibitor TMPyP4 Alters Adhesion and Migration of Breast-Cancer Cells MCF7 and MDA-MB-231
Source: Int J Mol Sci. 2019 May 30;20(11):2670. doi: 10.3390/ijms20112670 (PMC6600420; doi:10.3390/ijms20112670)
Supplement: Supplementary file 1 [file ijms-20-02670-s001.zip › ijms-491207 sp/Supplemetary file 2, adhesion, Figure 8.pptx]

## Slide 1
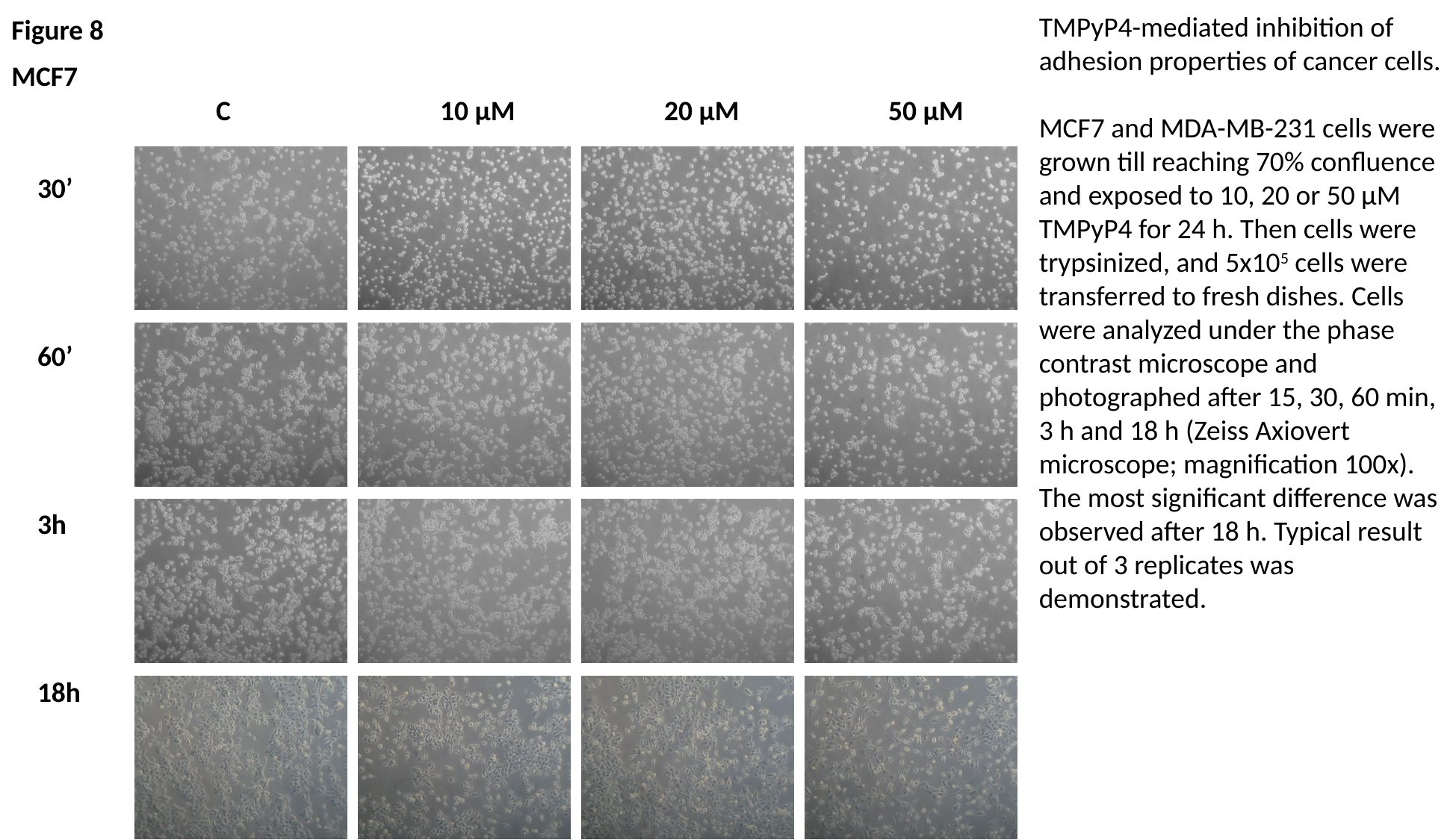

TMPyP4-mediated inhibition of adhesion properties of cancer cells.
MCF7 and MDA-MB-231 cells were grown till reaching 70% confluence and exposed to 10, 20 or 50 μM TMPyP4 for 24 h. Then cells were trypsinized, and 5x10^5^ cells were transferred to fresh dishes. Cells were analyzed under the phase contrast microscope and photographed after 15, 30, 60 min, 3 h and 18 h (Zeiss Axiovert microscope; magnification 100x). The most significant difference was observed after 18 h. Typical result out of 3 replicates was demonstrated.
Figure 8
MCF7
C		10 µM 		20 µM		50 µM
30’
60’
3h
18h

## Slide 2
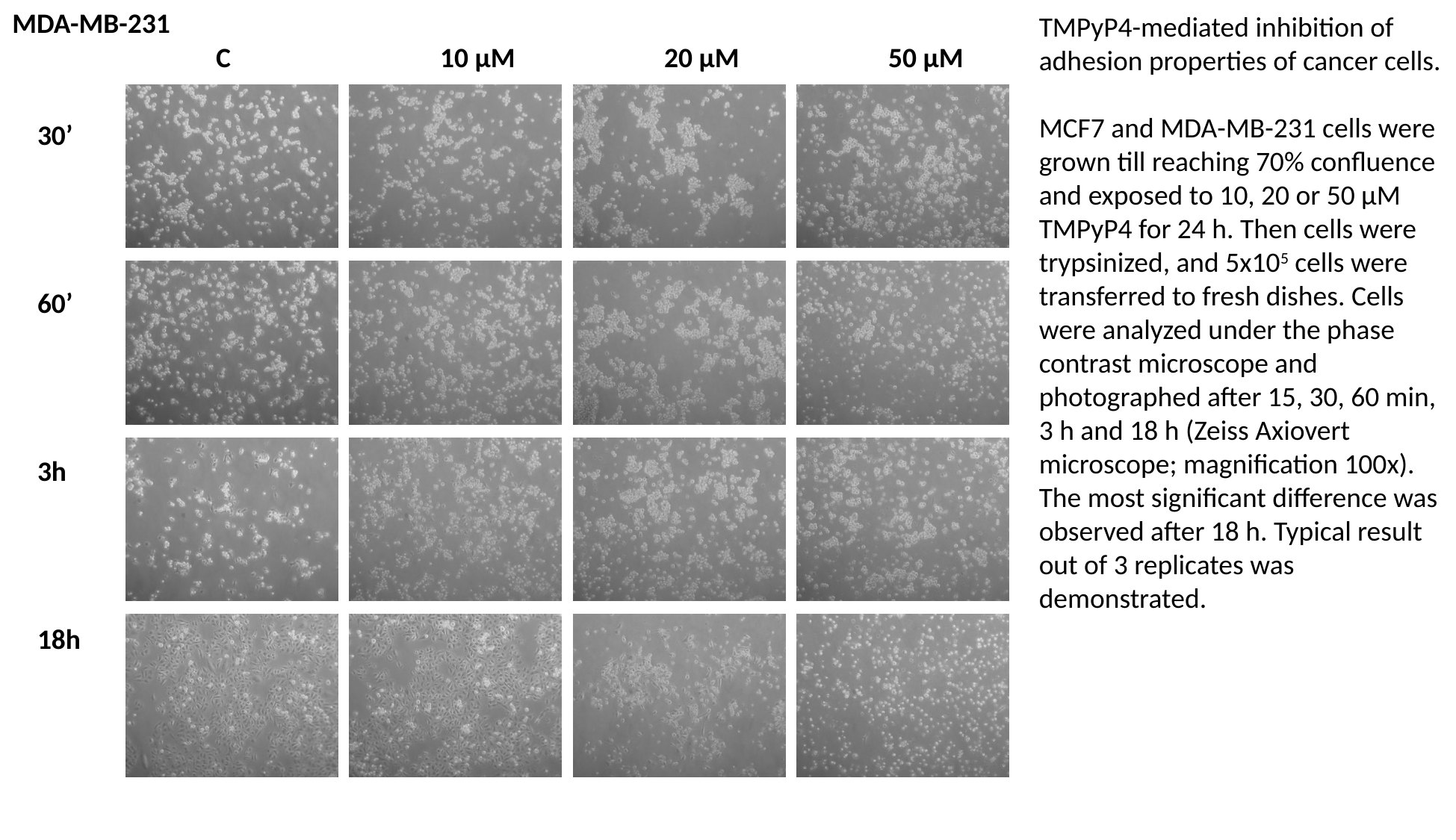

MDA-MB-231
TMPyP4-mediated inhibition of adhesion properties of cancer cells.
MCF7 and MDA-MB-231 cells were grown till reaching 70% confluence and exposed to 10, 20 or 50 μM TMPyP4 for 24 h. Then cells were trypsinized, and 5x10^5^ cells were transferred to fresh dishes. Cells were analyzed under the phase contrast microscope and photographed after 15, 30, 60 min, 3 h and 18 h (Zeiss Axiovert microscope; magnification 100x). The most significant difference was observed after 18 h. Typical result out of 3 replicates was demonstrated.
C		10 µM 		20 µM		50 µM
30’
60’
3h
18h
